# Cytoophidium assembly reflects upregulation of IMPDH activity

**DOI:** 10.1242/jcs.175265

**Published:** 2015-10-01

**Authors:** Chia-Chun Chang, Wei-Cheng Lin, Li-Mei Pai, Hsuan-Shu Lee, Shinn-Chih Wu, Shih-Torng Ding, Ji-Long Liu, Li-Ying Sung

**Affiliations:** 1 Institute of Biotechnology, National Taiwan University, Taipei 106, Taiwan, Republic of China; 2 Molecular Medicine Research Center, College of Medicine, Chang Gung University, Tao-Yuan 333, Taiwan, Republic of China; 3 Graduate Institute of Biomedical Science, College of Medicine, Chang Gung University, Tao-Yuan 333, Taiwan, Republic of China; 4 Department of Biochemistry, College of Medicine, Chang Gung University, Tao-Yuan 333, Taiwan, Republic of China; 5 Department of Internal Medicine, National Taiwan University Hospital, College of Medicine, National Taiwan University, Taipei 10051, Taiwan, Republic of China; 6 Department of Animal Science and Technology, National Taiwan University, Taipei 106, Taiwan, Republic of China; 7 Department of Physiology, Anatomy and Genetics, University of Oxford, Oxford OX1 3PT, UK; 8 Agricultural Biotechnology Research Center, Academia Sinica, Taipei 115, Taiwan, Republic of China

**Keywords:** CTPS, IMPDH, Cytoophidium, Nucleotide

## Abstract

Cytidine triphosphate synthase (CTPS) and inosine monophosphate dehydrogenase (IMPDH) (both of which have two isoforms) can form fiber-like subcellular structures termed ‘cytoophidia’ under certain circumstances in mammalian cells. Although it has been shown that filamentation of CTPS downregulates its activity by disturbing conformational changes, the activity of IMPDH within cytoophidia is still unclear. Most previous IMPDH cytoophidium studies were performed under conditions involving inhibitors that impair GTP synthesis. Here, we show that IMPDH forms cytoophidia without inhibition of GTP synthesis. First, we find that an elevated intracellular CTP concentration or treatment with 3′-deazauridine, a CTPS inhibitor, promotes IMPDH cytoophidium formation and increases the intracellular GTP pool size. Moreover, restriction of cell growth triggers the disassembly of IMPDH cytoophidia, implying that their presence is correlated with active cell metabolism. Finally, we show that the presence of IMPDH cytoophidia in mouse pancreatic islet cells might correlate with nutrient uptake in the animal. Collectively, our findings reveal that formation of IMPDH cytoophidia reflects upregulation of purine nucleotide synthesis, suggesting that the IMPDH cytoophidium plays a role distinct from that of the CTPS cytoophidium in controlling intracellular nucleotide homeostasis.

## Abstract (precis)

**Summary:** The formation of IMPDH cytoophidia reflects upregulation of purine nucleotide synthesis, suggesting that the IMPDH cytoophidium plays a distinct role from that of the CTPS cytoophidium in controlling nucleotide homeostasis.

## INTRODUCTION

Nucleotides are not only essential for DNA and RNA synthesis, but are also involved in various metabolic processes and signaling transductions. Inosine monophosphate dehydrogenase (IMPDH) and cytidine triphosphate synthase (CTPS) (both of which have two isoforms in mammals) catalyze the rate-limiting steps of *de novo* GTP and CTP synthesis, respectively (Lieberman, 1956; Yoshioka et al., 1992).

Recently, several studies have demonstrated that CTPS and IMPDH are involved in the formation of a subcellular fiber-like structure termed ‘rods and rings’ or the ‘cytoophidium’ (Carcamo et al., 2011; Ingerson-Mahar et al., 2010; Ji et al., 2006; Liu, 2010; Noree et al., 2014, 2010). This structure is not membrane-bound and is not associated with any known organelle in mammalian cells (Thomas et al., 2012). Recently, CTPS-based, IMPDH-based and mixed cytoophidia have been found in mammalian cells. Different types of cytoophidia displayed similar filamentous morphology, but the proportions of them changed with various inductions; the IMPDH inhibitors mycophenolic acid (MPA) and Ribavirin induce only IMPDH-based cytoophidia, whereas 6-diazo-5-oxo-L-norleucine (DON), which interrupts purine and pyrimidine biosynthesis, triggers filamentation of both enzymes (Keppeke et al., 2015). These results suggest that formation of CTPS and IMPDH cytoophidia can be regulated independently.

Multiple inhibitors of nucleotide synthesis have been applied in studies of the characteristics of IMPDH cytoophidia. Overexpression of fluorescent fusion protein approaches have also been adopted in some studies (Carcamo et al., 2011; Ji et al., 2006; Thomas et al., 2012). However, information about the dynamics of non-inhibitory treatment that induces IMPDH cytoophidium is required to discover more about their physiological function and regulation.

Herein, we aimed to investigate the regulation of the IMPDH cytoophidium and their putative role in mammalian cell metabolism. We show that regulation of the IMPDH cytoophidium is different from that of the CTPS cytoophidium. Overproduction of CTP or inhibition of CTP synthesis stimulated IMPDH cytoophidium assembly along with an increase of intracellular GTP. We also found that IMPDH cytoophidia spontaneously form in mouse BNL CL2 cells. Maintenance of these cytoophidia is correlated with active cell proliferation. Finally, we show that the presence of IMPDH cytoophidia in mouse pancreatic islet cells might correlate with nutrient uptake of the animal. Our findings indicate that IMPDH tends to form cytoophidia when *de novo* purine synthesis is positively regulated. They also provide new insights into nucleotide metabolism and provide a basis for further research into the potential of the IMPDH cytoophidium to be a biomarker or drug target in clinical applications.

## RESULTS AND DISCUSSION

### CTPS and IMPDH form two types of cytoophidia

**Fig. 1.**
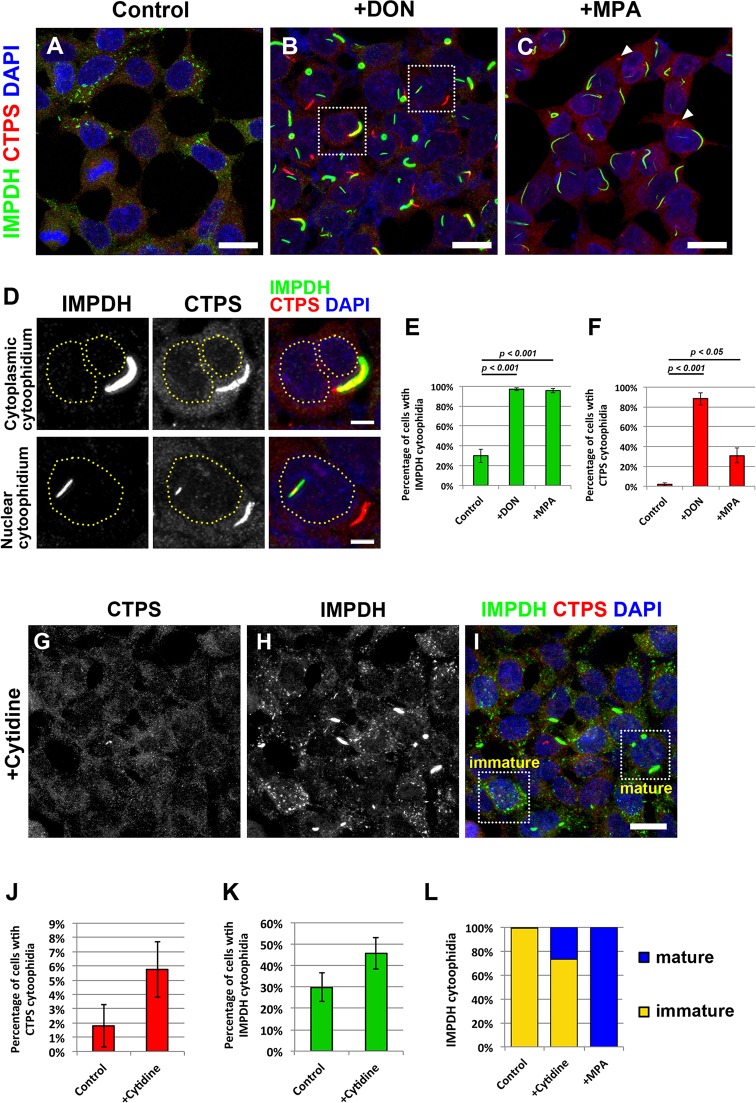
**CTPS and IMPDH can form independent cytoophidium structures.** Immunofluorescence of (A) untreated HEK 293T cells, and (B) HEK 293T cells treated with DON (10 μg/ml) or (C) MPA (10 μM) for 1 day before fixation. Arrowheads indicate CTPS cytoophidia. Scale bars: 20 μm. (D) Magnified views of the boxed areas in B showing colocalization of CTPS and IMPDH cytoophidia with the cytoplasm and the nucleus. Dashed lines indicate the outline of the nuclei. Scale bar: 5 μm. (E,F) Mean±s.e.m. percentages of cells with IMPDH and CTPS cytoophidia. *P*-values were calculated with a Student's *t*-test. (G–I) Immunofluorescence of HEK 293T cells cultured in medium with 100 μM cytidine for 1 h before fixation. Mature and immature IMPDH cytoophidia are shown in the selected areas in I. (J,K) Mean±s.e.m. percentages of cells with CTPS and IMPDH cytoophidia. There is no significant difference between the control and cytidine-treated group (Student's *t*-test, *P* values=0.16 and 0.19). (L) Ratios of mature and immature IMPDH cytoophidia observed in cells cultured under normal culture conditions (control), treated with 100 μM cytidine for 1 h (+cytidine), and treated with 10 μM MPA for 1 day (+MPA).

CTPS and IMPDH have been identified as the main components of cytoophidia in mammalian cells (Carcamo et al., 2011; Chen et al., 2011). As shown in a recent study, CTPS and IMPDH can form two independent types of cytoophidium structure (Keppeke et al., 2015). To determine whether CTPS and IMPDH cytoophidia are co-regulated or respond to different stimuli, we first cultured human HEK 293T cells in medium containing a glutamine analog, DON, which blocks CTP and GTP biosynthesis, or an IMPDH specific inhibitor, MPA. Both DON and MPA can induce cytoophidium assembly (Carcamo et al., 2011; Chen et al., 2011; Ji et al., 2006; Keppeke et al., 2015). Under normal culture conditions, IMPDH cytoophidia were observed in ∼30% of cells, whereas CTPS cytoophidia were hardly detectable (Fig. 1A,E,F). After treatment with DON for 1 day, the number of cells with CTPS and IMPDH cytoophidia significantly increased to over 80% and 90% of cells, respectively (Fig. 1B,E,F). CTPS and IMPDH cytoophidia were observed as separate structures, but sometimes fully or partially colocalized with the cytoplasm and the nucleus (Fig. 1B,D). When cells were treated with MPA, IMPDH cytoophidia formed in more than 90% of cells. Under the same conditions, more than 20% of cells also expressed CTPS cytoophidia (Fig. 1C,E,F). In our previous work, we established a stably transfected CTPS1–GFP HEK 293T cell line (CTPS1-overexpressing cells) (Aughey et al., 2014). It has been shown that CTPS tends to aggregate *in vitro* when CTP and CTPS concentrations are high (Barry et al., 2014). Therefore, a fivefold increase in CTPS1 protein level resulted in the induction of large CTPS cytoophidia in more than 80% of CTPS1-overexpressing cells (supplementary material Fig. S1B–F). We then performed immunostaining on CTPS1-overexpressing cells to check whether regulation of IMPDH cytoophidia was affected by CTPS1 overexpression. Interestingly, a significant increase in cells with IMPDH cytoophidia was found for this cell line, up to more than 60%, with no changes in the IMPDH expression level (supplementary material Fig. S1A,C–F). Similar to DON-induced cytoophidia, some IMPDH cytoophidia partially colocalized with CTPS cytoophidia. This was also the case in cells transfected with a construct encoding CTPS1 protein without the GFP tag, excluding the possibility of a side effect caused by chimeric recombinant protein (supplementary material Fig. S1H–M). Furthermore, nucleotide analysis showed that the GTP level was not changed in CTPS1-overexpressing cells, indicating that these IMPDH cytoophidia were not induced by a GTP-deficient cell status (supplementary material Fig. S1G), which has been suggested to be the condition that stimulates formation of IMPDH cytoophidium (Calise et al., 2014). This result raised the possibility that the 40% increase in the CTP level in CTPS1-overexpressing cells promotes IMPDH cytoophidium assembly. We then increased the intracellular CTP pool by supplementation of cytidine, which can be converted into CTP through a salvage pathway, and found that the percentage of cells with IMPDH cytoophidia slightly increased (Fig. 1G–K). As reported previously, the cytoophidium could generally be divided into ‘mature’ and ‘immature’ cytoophidium by their appearance (Calise et al., 2014). Several punctate and small spicule-shaped immature cytoophidia can form within a single cell, and they can undergo serial fusions to form larger cytoophidia (Gou et al., 2014; Thomas et al., 2012). By contrast, only one or a few large linear or ring-shaped mature cytoophidia were present in one cell in most cases (Fig. 1I). Under treatment with cytidine, more cells were found with mature IMPDH cytoophidia, and these were widely found when cells were treated with inhibitors blocking GTP synthesis (Fig. 1L).

### Inhibition of *de novo* CTP synthesis promotes IMPDH cytoophidium formation

**Fig. 2.**
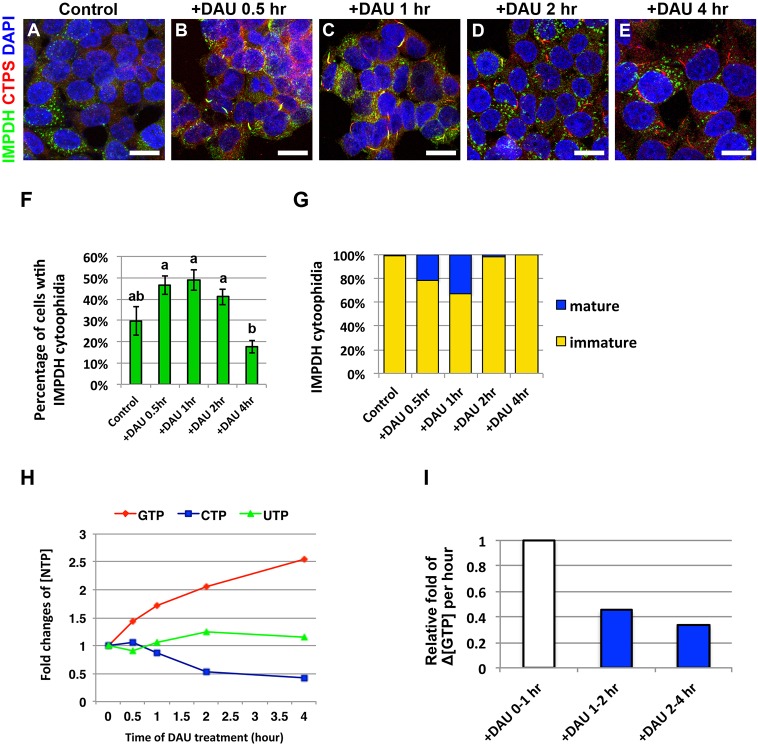
**Inhibition of *de novo* CTP synthesis promotes IMPDH cytoophidium assembly.** (A–E) Immunofluorescence of HEK 293T cells treated with 100 μM DAU for 0, 0.5, 1, 2 and 4 h. Scale bars: 20 μm. (F) Mean±s.e.m. percentages of cells with IMPDH cytoophidia. Lowercase letters represent significant differences between groups (one-way ANOVA with Tukey's test). (G) Ratios of mature and immature IMPDH cytoophidia observed in cells cultured in 100 μM DAU for different durations. (H) Nucleotide levels in cells treated with 100 μM DAU for different durations. Nucleotide concentrations for each point were normalized to the level of ATP. (I) The changes of GTP concentration within 1 h intervals shows that intracellular GTP accumulates faster in the first hour than later few hours of DAU treatment.

Purine and pyrimidine biosynthetic pathways are reciprocally regulated through sharing the pool of phosphoribosyl pyrophosphate (PRPP), which is the common substrate of nucleotide synthetic pathways. CTP functions as a negative regulator of multiple steps in the *de novo* pyrimidine synthetic pathway (Lipscomb, 1994; Long and Pardee, 1967). We speculated that the inhibition of pyrimidine biosynthesis caused by an elevated CTP level, which activates *de novo* purine synthesis, promotes IMPDH cytoophidium assembly. Thus, we next treated HEK 293T cells with 3′-deazauridine (DAU), an analog of uridine that can inhibit CTP biosynthesis by competitive inhibition of CTPS (Moriconi et al., 1986). During the first 1-h period of DAU treatment, the percentages of cells with IMPDH cytoophidia gradually increased, and more mature IMPDH cytoophidia were also observed (Fig. 2A–C,F,G). However, the numbers of cells with IMPDH cytoophidia declined after prolonged DAU treatment. After 4 h of DAU treatment, less than 20% of cells had immature IMPDH cytoophidia, and mature IMPDH cytoophidium were barely detectable (Fig. 2D–G). Meanwhile, the number of CTPS cytoophidia gradually increased under the same conditions (Fig. 2A–E). To check whether IMPDH cytoophidium formation correlated with increased GTP synthesis, we traced nucleotide levels within the period of DAU treatment. The result shows that whereas the CTP level had gradually decreased to less than half of the original level, the GTP level had doubled at the point of 2-h DAU treatment (Fig. 2H). Additionally, the rate for GTP accumulation in the initial 1 h was more than twice the rate in the later few hours of DAU treatment (Fig. 2I). It is known that guanine nucleotides inhibit multiple enzymes in the *de novo* GTP synthetic pathway including PRPP synthetase, glutamine-PRPP amidotransferase and IMPDH (Lehninger et al., 2013). This provides the possible explanation for the decrease of IMPDH cytoophidia and the gradually slowing accumulation of GTP during 1 to 4 h of DAU treatment. We also subjected HeLa cells to the same conditions, and observed a similar phenomenon (supplementary material Fig. S2). These results indicate that inhibition of CTP synthesis with DAU activates purine nucleotide synthesis in human cells and promotes IMPDH cytoophidia assembly.

### Inhibition of cell growth triggers disassembly of IMPDH cytoophidia

**Fig. 3.**
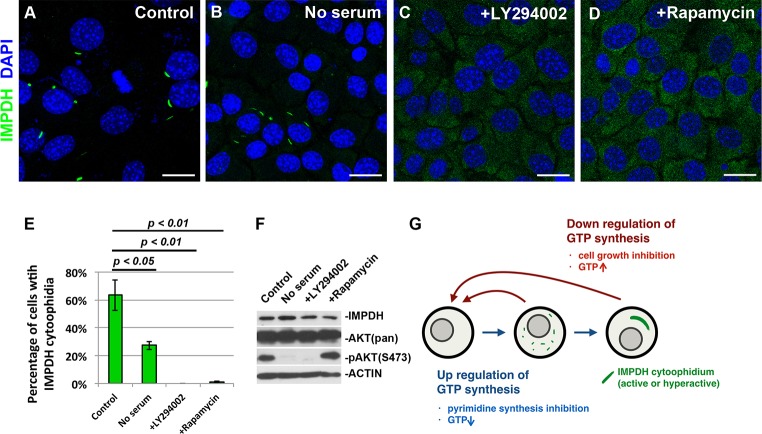
**IMPDH forms cytoophidia in specific cell types *in vitro*.** (A–D) Immunofluorescence of mouse BNL CL2 cells that were untreated (control), cultured with serum-free medium for 1 day, or treated with 50 μM LY294002 (PI3K inhibitor) or 1 μM rapamycin (mTOR inhibitor) for 6 h before fixation. Scale bars: 20 μm. (E) Mean±s.e.m. percentages of cells with IMPDH cytoophidia after culture under various conditions. *P*-values were calculated with a Student's *t*-test. (F) Immunoblotting of BNL CL2 cells cultured under various conditions. AKT(pan), total ATK; pAKT, phosphorylated AKT. (G) A model for the regulation of IMPDH cytoophidium assembly.

In a screening of mammalian cell lines, we found that mature IMPDH cytoophidia were normally expressed in the mouse fetal liver cell line BNL CL2. Under normal culture conditions, IMPDH cytoophidia spontaneously formed in ∼60% of BNL CL2 cells (Fig. 3A). However, only 22% (27/121) of cells undergoing mitosis contained detectable IMPDH cytoophidia. When cells launch division, they have to increase nucleotide production before or during S phase to meet the needs of protein expression and DNA replication. To test the correlation of IMPDH cytoophidia and cell growth, we cultured BNL CL2 cells in serum-free medium for 1 day. The percentage of cells with IMPDH cytoophidia dropped to only 20%, suggesting that active signaling pathways for cell growth are important for maintaining the IMPDH cytoophidium structure (Fig. 3B,E). Moreover, we also treated BNL CL2 cells with the phosphoinositide 3-kinase (PI3K) inhibitor LY294002 and rapamycin, a mammalian target of rapamycin (mTOR) inhibitor, in order to restrict cell growth by blocking the PI3K–AKT–mTOR pathway. Inhibition of the PI3K–AKT–mTOR pathway was confirmed by the decreased level of phosphorylation at S473 of AKT, a marker of active PI3K signaling (Fig. 3F). The disturbance of cell cycle progression by inhibitors was also confirmed by flow cytometry (supplementary material Fig. S3A). Consistent with the above results, IMPDH cytoophidia were barely observed upon inhibition of the pathway, whereas the IMPDH protein expression level did not remarkably change (Fig. 3C–F). Similar results were observed in HEK 293T cells. When HEK 293T cells were cultured in medium containing LY294002 or rapamycin for 6 h, the percentages of cells with IMPDH cytoophidia significantly dropped, and additional DAU in the last 1 h of treatments failed to stimulate IMPDH cytoophidium assembly (supplementary material Fig. S3B–F). These results again support our hypothesis that upregulation of purine nucleotide synthesis promotes IMPDH cytoophidium assembly (Fig. 3G).

### IMPDH forms cytoophidia in mouse pancreatic islet cells

**Fig. 4.**
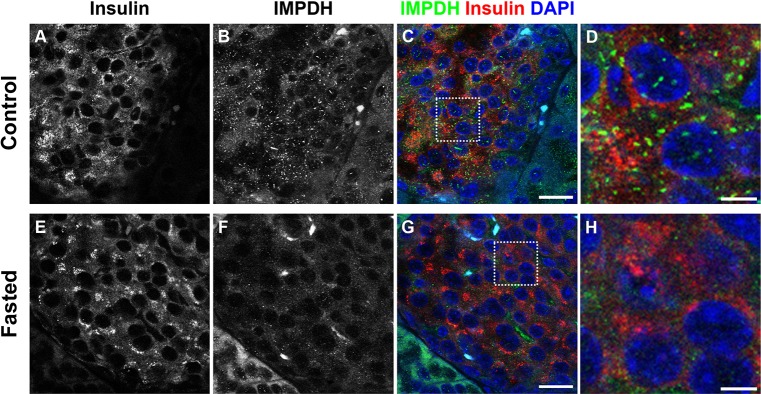
**IMPDH forms cytoophidia in mouse pancreatic islet cells.** (A–C) Immunofluorescence on a normal mouse pancreas section showing abundant immature IMPDH cytoophidia in islet cells. Scale bar: 20 μm. (D) Magnified view of boxed area in C. Scale bar: 5 μm. (E–G) Immunofluorescence on a section of pancreas from a mouse fasted overnight shows that the number of IMPDH cytoophidia in islet cells decreased. Scale bar: 20 μm. (H) Magnified view of boxed area in G. Scale bar: 5 μm.

To determine whether IMDPH forms cytoophidia *in vivo*, we used an antibody against IMPDH to stain sections of various mouse tissues, including intestine, stomach, skin, thymus, lymph node, brain, liver, testis and pancreas. We found that IMPDH cytoophidia were detectable in only a few cells in most tissues, except in the pancreas, where we could detect very abundant IMPDH cytoophidia. When co-stained with the anti-insulin antibody, IMPDH cytoophidia were observed in many β cells (Fig. 4A–D). The secretion of insulin is tightly controlled by a combination of metabolic-coupling factors; for example, insulin-exocytosis-associated GTPase enzymes utilize GTP to promote insulin secretion, and an increased GTP level is found at high glucose concentration (Robertson et al., 1991; Zou et al., 2014). Other studies have shown that treatment with MPA or ribavirin impedes the release of insulin in rat islets, and that this inhibition can be eliminated by guanine supplementation, indicating that sufficient GTP is essential for insulin secretion (Kowluru et al., 1996; Metz et al., 1992). Accordingly, we propose that IMPDH filamentation in pancreatic β cells is correlated with insulin secretion. To confirm this, we performed immunostaining on tissues sections of pancreas collected from mice that were fasted overnight (18 h) to keep their blood glucose concentration low. Consequently, the number of IMPDH cytoophidia reduced in most islets of fasted mice (Fig. 4E–H). We also performed whole-mount staining on pancreatic islets freshly isolated from mice. Consistent with the above results, many IMPDH cytoophidia were observed in freshly isolated islets (supplementary material Fig. S4A–C). To reduce glucose-stimulated insulin secretion, we further cultured isolated islets in Hank's balanced salt solution (HBSS), as a nutrient-deficient condition, for 3 h prior to fixation. As expect, disassembly of IMPDH cytoophidia was apparent in most islet cells (supplementary material Fig. S4D–F). These results show that the regulation of IMPDH cytoophidium in pancreatic islet might respond to the nutrient uptake of the cell.

Most previous IMPDH cytoophidium studies have been performed with IMPDH inhibitors or glutamine analogs. Given that such inhibitors directly disturb GTP biosynthesis, it is hard to determine the real regulation and function of the IMPDH cytoophidium under those conditions. Here, we show that assembly of the IMPDH cytoophidium is promoted by active regulation of purine synthesis in mammalian cell lines. A previous structural study has demonstrated that filamentation of human IMPDH1 protein occurs in the presence of its effector MgATP (Labesse et al., 2013). This is consistent with our findings. Although most of the previous descriptions about IMPDH cytoophidium have been focused on IMPDH2, the possibility of cross-reaction between two IMPDH isoforms cannot be excluded due to their high sequence similarity (Calise et al., 2014; Keppeke et al., 2015; Natsumeda et al., 1990). In this study, our immunostaining results were acquired with a mouse monoclonal antibody for human IMPDH1 (ab55297, Abcam), with two other anti-human IMPDH1 polyclonal antibodies (ab84957 and ab89048, Abcam) also tested. Because staining with the three antibodies resulted in an identical pattern, we cannot rule out the possibility that both isoforms were under our observation.

It has been shown that CTPS cytoophidium acts as a negative regulator of CTP synthesis (Barry et al., 2014). Therefore, IMPDH and CTPS cytoophidia might play distinct roles in coordinating the balance of nucleotide pools. Further research is required to reveal how cytoophidium affects the enzymatic activity of IMPDH. Given that IMPDH and CTPS have been considered as drug targets in the case of several types of disease, our results not only provide valuable information for understanding the putative roles of cytoophidia in cell metabolism, but also support future studies to determine the potential of the cytoophidium as a biomarker or target in medical applications or treatments.

## MATERIALS AND METHODS

### Cell culture

HEK 293T cells, HeLa cells and mouse BNL CL2 cells were cultured in Dulbecco's modified Eagle's medium (DMEM) with high glucose (11965, Gibco) supplemented with 10% fetal bovine serum (04-001, Biological Industries) and 1% penicillin-streptomycin (15140, Gibco). Cells were kept in a 37°C incubator with 5% CO_2_. DON (D2141, Sigma-Aldrich) and cytidine were dissolved in water. Mycophenolic acid (M3536, Sigma-Aldrich), guanosine, 3′-deazauridine (sc-394445, Santa Cruz Biotechnology), LY294002 (9901S, Cell Signaling) and rapamycin (R0395, Sigma-Aldrich) were dissolved in DMSO (D2650, Sigma-Aldrich). Chemicals were added in culture medium as described.

### Tissue preparation

Animal maintenance and the procedures described herein were approved by the Institutional Animal Care and Use Committee of National Taiwan University. Tissues for paraffin embedding were collected from 20-week-old male ICR mice and then fixed with 4% paraformaldehyde (43368, Alfa Aesar) overnight before proceeding of standard embedding protocol. For whole-mount staining, pancreatic islets were isolated from 20-week-old male ICR mice with the method described (Neuman et al., 2014).

### Immunofluorescence

Cells were cultured on glass cover slides and fixed with 4% paraformaldehyde in PBS for 10 min. Fixed cells, mouse islets and tissue sections were incubated in primary antibody in PBS with 2% bovine serum albumin (BSA, A9647, Sigma-Aldrich) and 0.2% Triton X-100 (X100, Sigma-Aldrich) for at least 2 h at room temperature. After washing with PBS, samples were incubated in secondary antibody, which is diluted in the same solution as used in the primary antibody dilution. At least 2 h after the secondary antibody reaction, samples were washed and mounted with PBS. Antibodies used in this study include: rabbit anti-human-CTPS1 IgG (1:500, GTX105265, GeneTex), mouse anti-human-IMPDH1 IgG (1:500, ab55297, Abcam) and rabbit anti-insulin antibody (1:500, 4590, Cell Signaling). Alexa-Fluor-488-conjugated goat anti-mouse-IgG (1:500, A11029, Invitrogen) and Alexa-Fluor-647-conjugated goat anti-rabbit-IgG (1:500, A21244, Invitrogen) were used.

### Microscopy

Images were acquired under the 63× objective of a laser-scanning confocal microscope (Leica TCS SP5 II confocal microscope).

### Immunoblotting

Cell lysates were prepared with RIPA lysis buffer (20-188, Millipore) and were run on a 12% polyacrylamide gel. PVDF membranes (GE Healthcare) were used for protein transfer. The membrane was incubated with 5% milk in PBS at room temperature for 1 hour for blocking, followed by overnight incubation with primary antibodies at 4°C. After primary antibody reaction, the membrane was washed with PBST three times, followed by further incubation with secondary antibodies at room temperature for 2 hours. After three times washing with PBST, the signals of secondary antibodies were detected using ECL (WBLUF0500, Millipore) and X-ray film. Antibodies used for western blotting include: rabbit anti-human CTPS1 IgG (1:3000, GTX105265, GeneTex), mouse anti-human IMPDH1 IgG (1:1000, ab55297, Abcam), rabbit-anti Akt (1:1000, 4691S, Cell Signaling), rabbit anti-pS473 Akt (1:1000, 4060S, Cell Signaling) and mouse anti-β-actin (1:10,000, A5441, Sigma-Aldrich) antibodies. HRP-conjugated goat anti-mouse-IgG (1:3000, ab6789, Abcam) and HRP-conjugated goat anti-rabbit-IgG (1:3000, 31460, Thermo).

### Nucleotide analysis

Quantification of intracellular nucleotides was performed following the method in Aughey et al. (2014). In brief, 7×10^6^ cells were lysed in 80% methanol. After centrifugation at 13,000 ***g*** for 10 min, the supernatants were collected and dried. Pellets were resuspended in water and analyzed using Acquity Ultra Performance Liquid Chromatography (UPLC, Waters) interfaced with a PDA photodiode array (Waters).

### Statistics

Statistical analysis was performed using unpaired two-tailed Student's *t*-tests or one-way ANOVA and Tukey's test. The quantification of the percentage of cells with cytoophidia was from at least three repeat experiments, and more than 100 cells were counted for each quantification. All error bars shown in graphs represent s.e.m.
